# Supplementary figures and images for: The Rab7-Epg5 and Rab39-ema modules cooperatively position autophagosomes for efficient lysosomal fusions
Source: eLife. 2025 Oct 28;13:RP102663. doi: 10.7554/eLife.102663 (PMC12563567; doi:10.7554/eLife.102663)

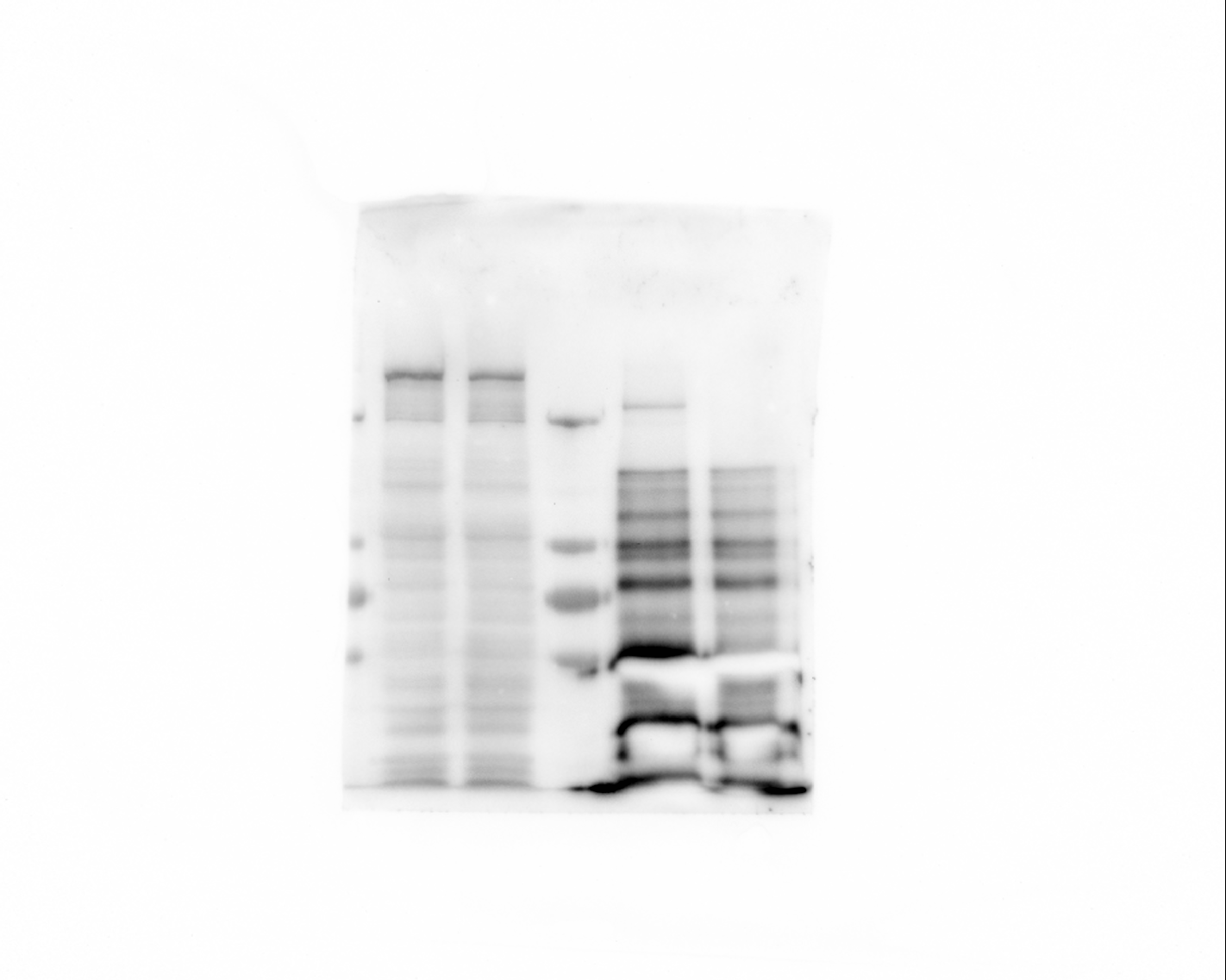

Supplement: Figure 5—source data 1. [file elife-102663-fig5-data1.zip › lab_2024-05-24_17h14m41s(Chemiluminescence)_a-Dhc2_inverz.tif]

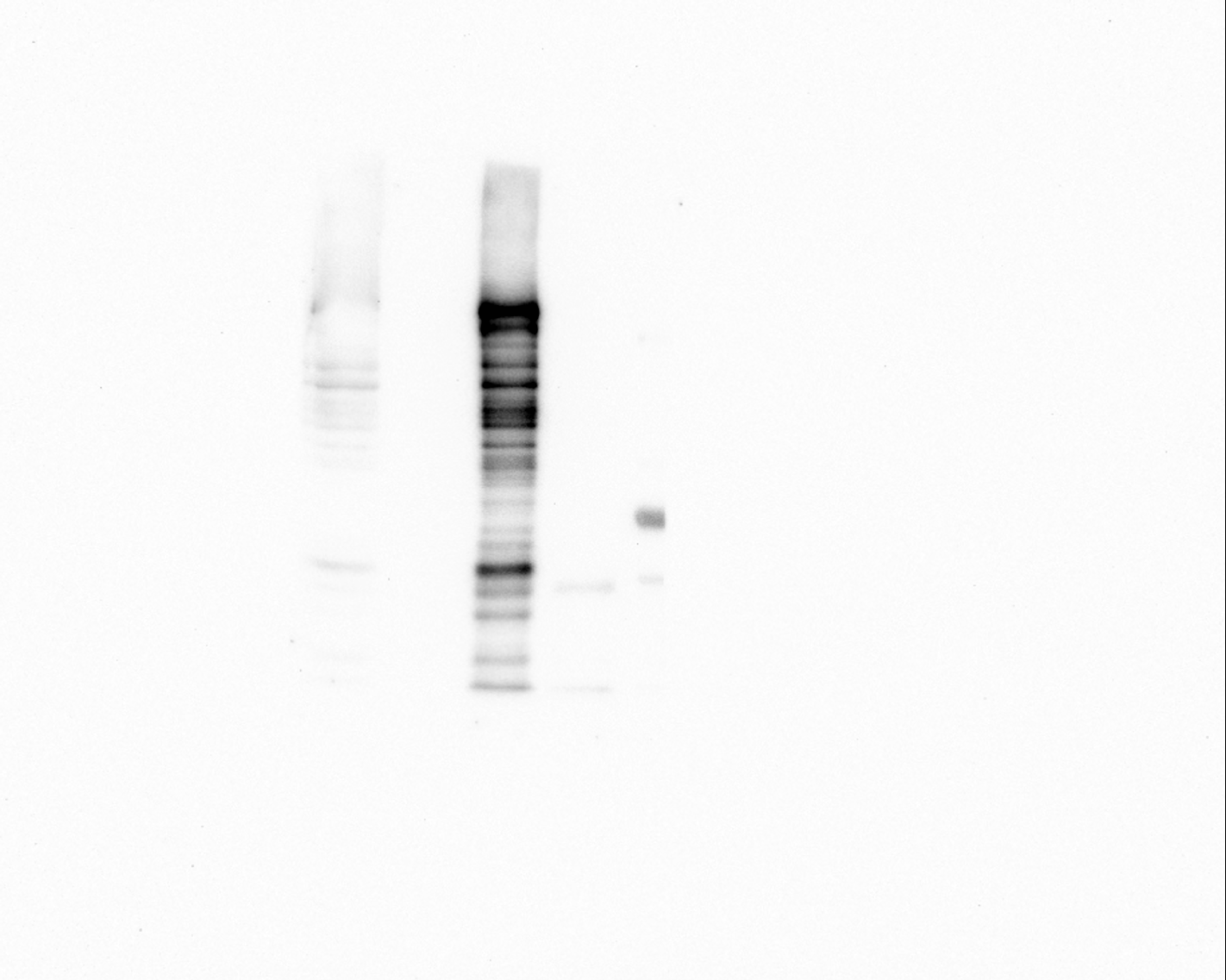

Supplement: Figure 5—source data 1. [file elife-102663-fig5-data1.zip › lab_2024-06-21_14h41m11s(Chemiluminescence)_a-HA_Ib_inverz.tif]

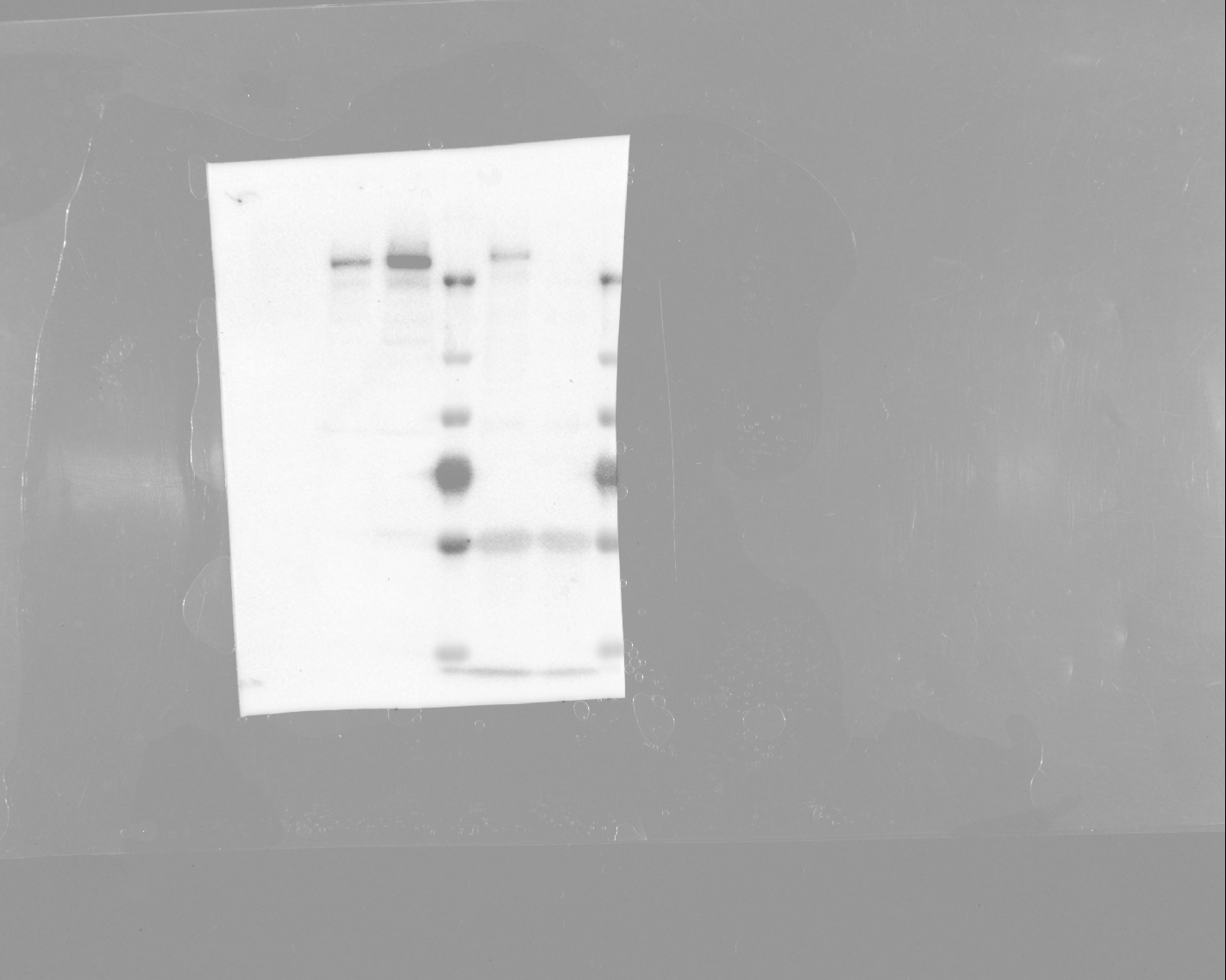

Supplement: Figure 5—source data 1. [file elife-102663-fig5-data1.zip › lab_2024-07-12_16h25m14s(Composite)_a-HA_inverz.tif]

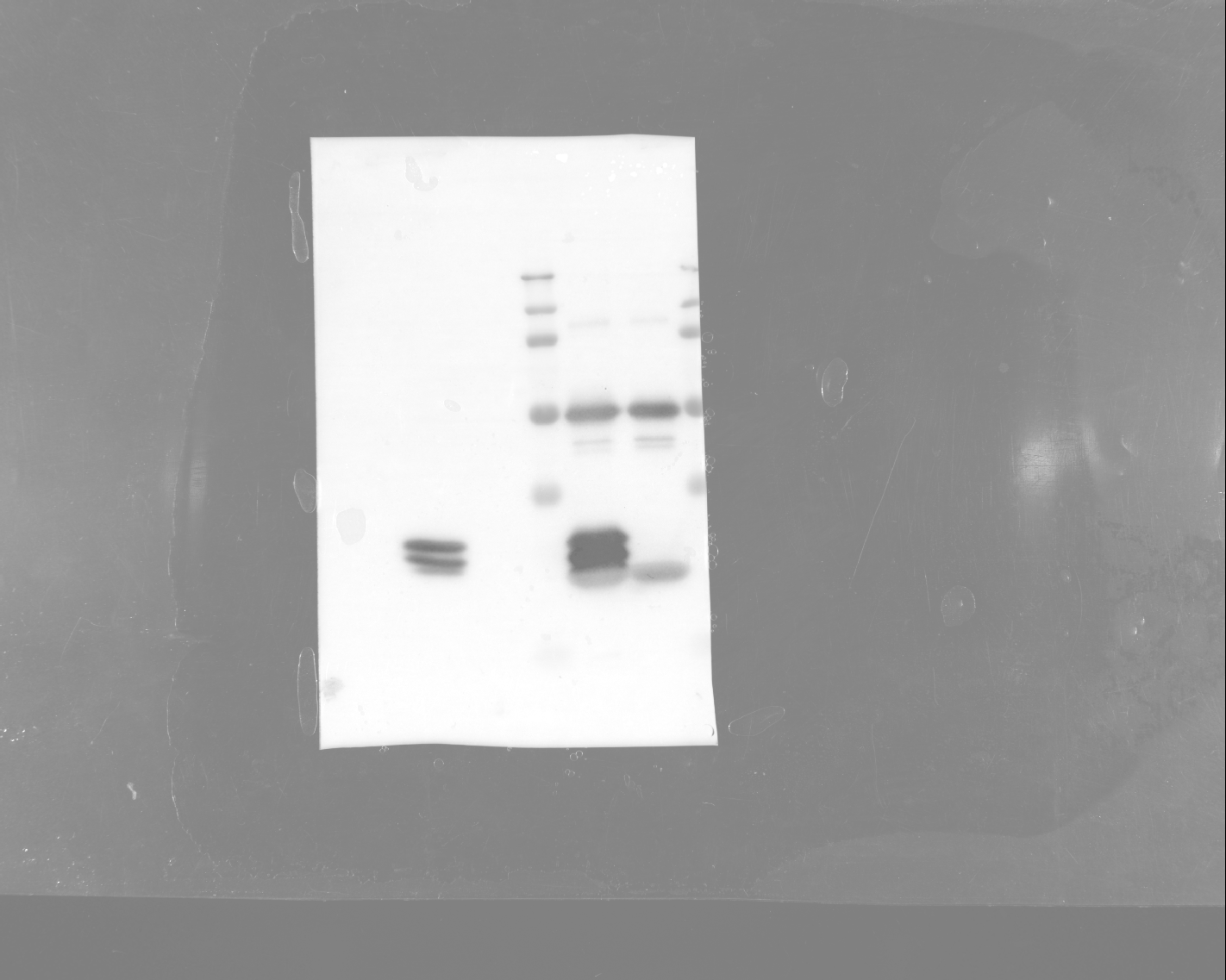

Supplement: Figure 5—source data 1. [file elife-102663-fig5-data1.zip › lab_2024-07-16_16h39m43s(Composite)_a-FLAG2inverz.tif]

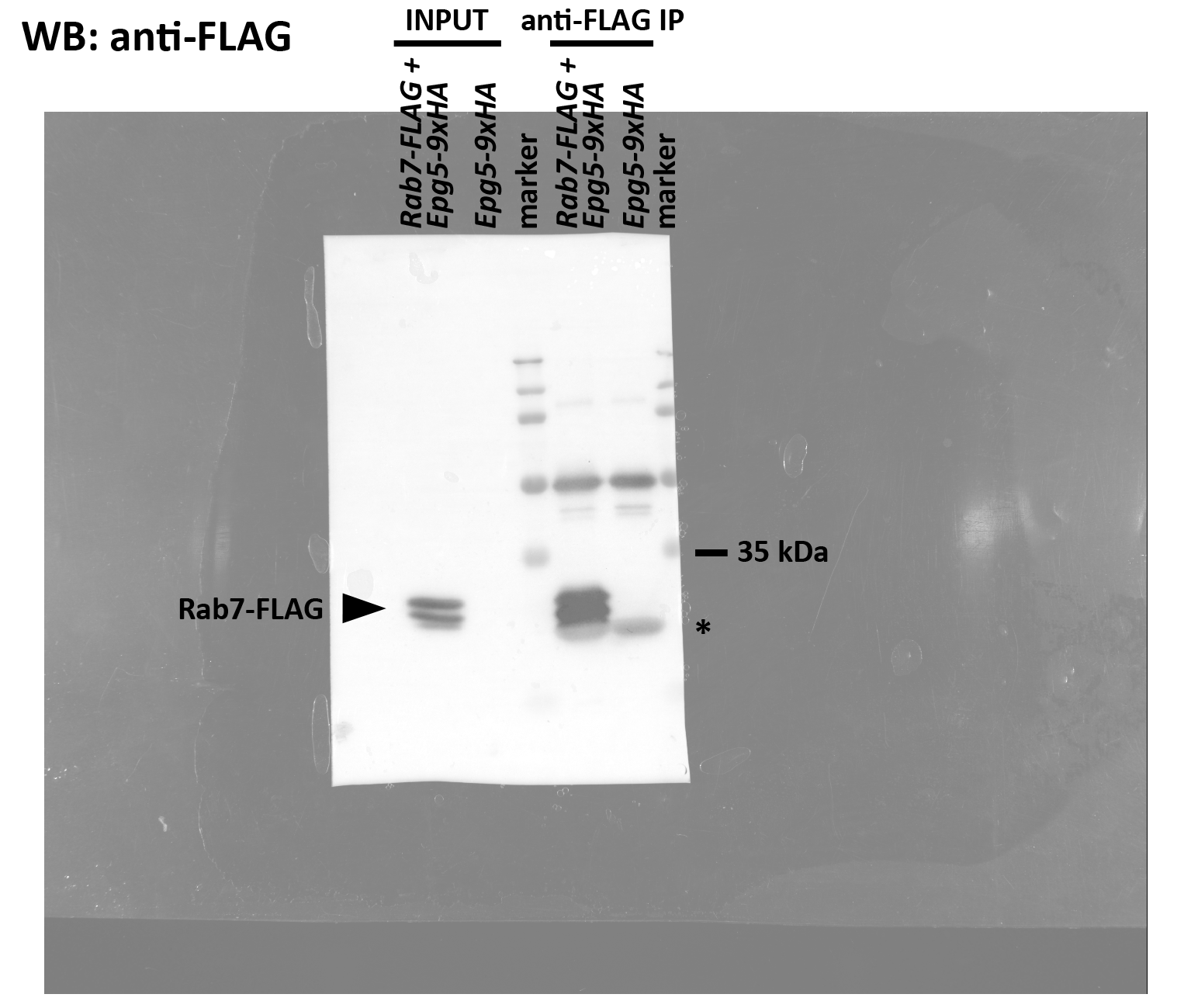

Supplement: Figure 5—source data 2. [file elife-102663-fig5-data2.zip › 5L_anti-FLAG_raw_labelled.tif]

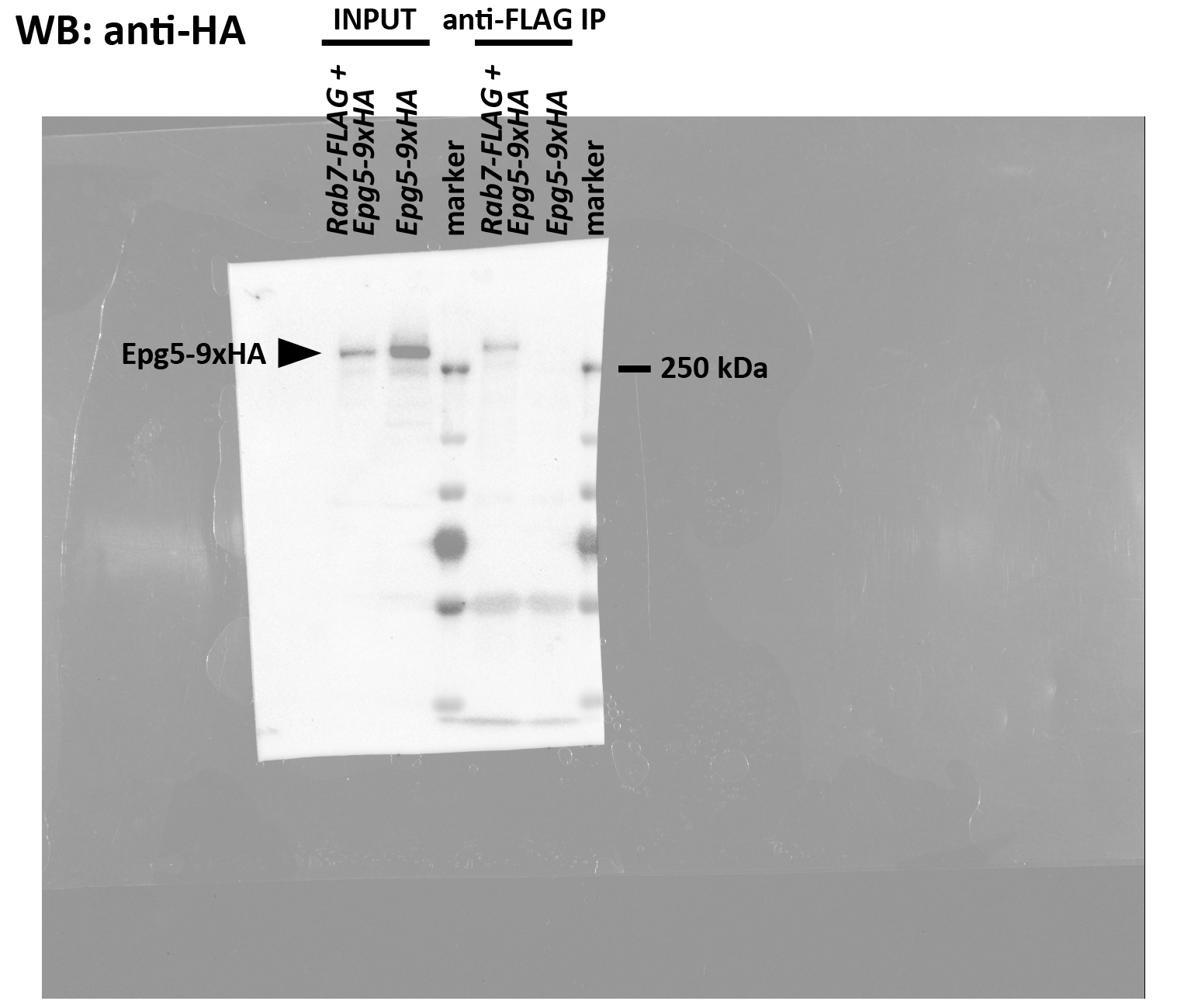

Supplement: Figure 5—source data 2. [file elife-102663-fig5-data2.zip › 5L_anti-HA_raw_labelled.tif]

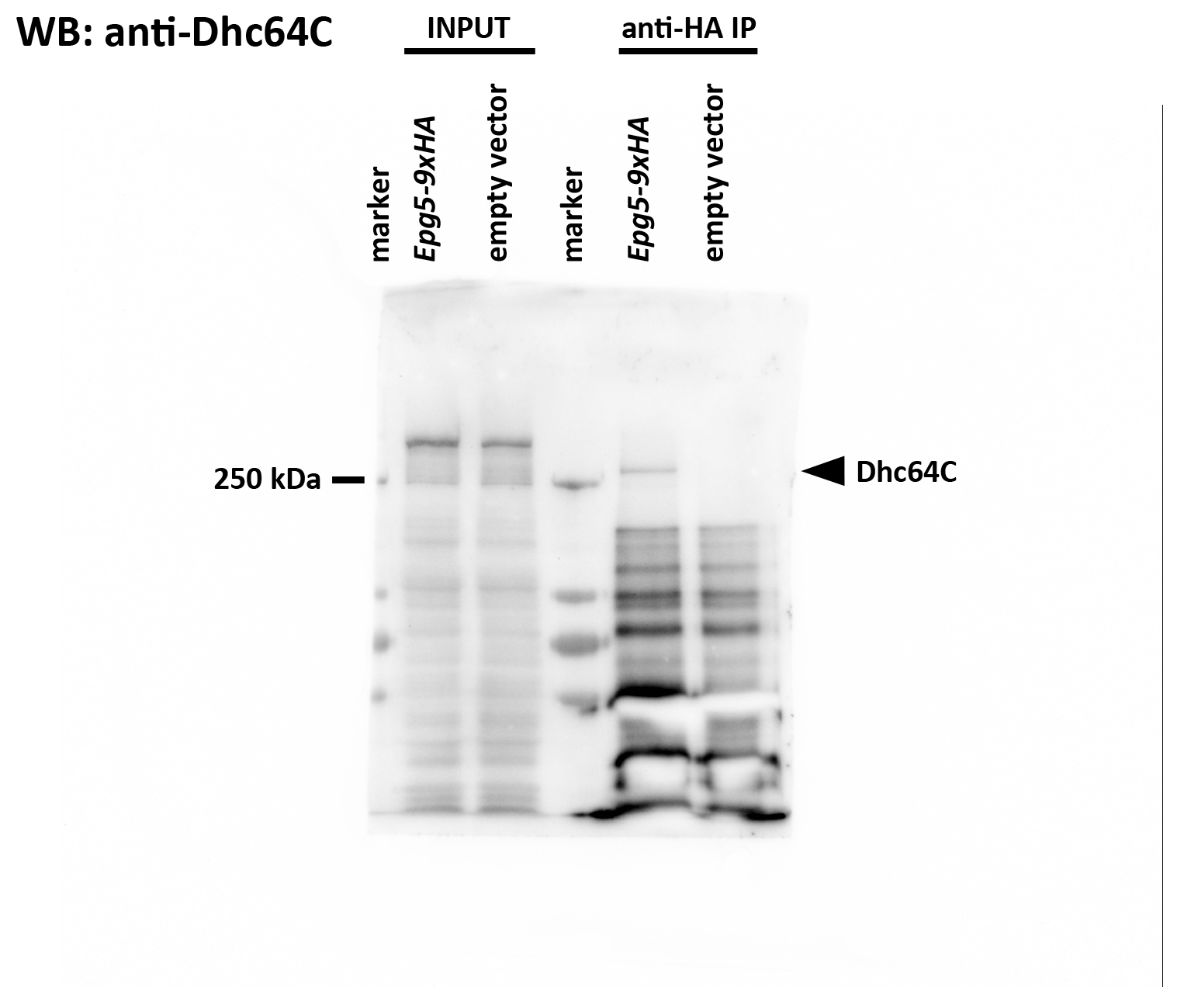

Supplement: Figure 5—source data 2. [file elife-102663-fig5-data2.zip › 5M_anti-Dhc64C_raw_labelled.tif]

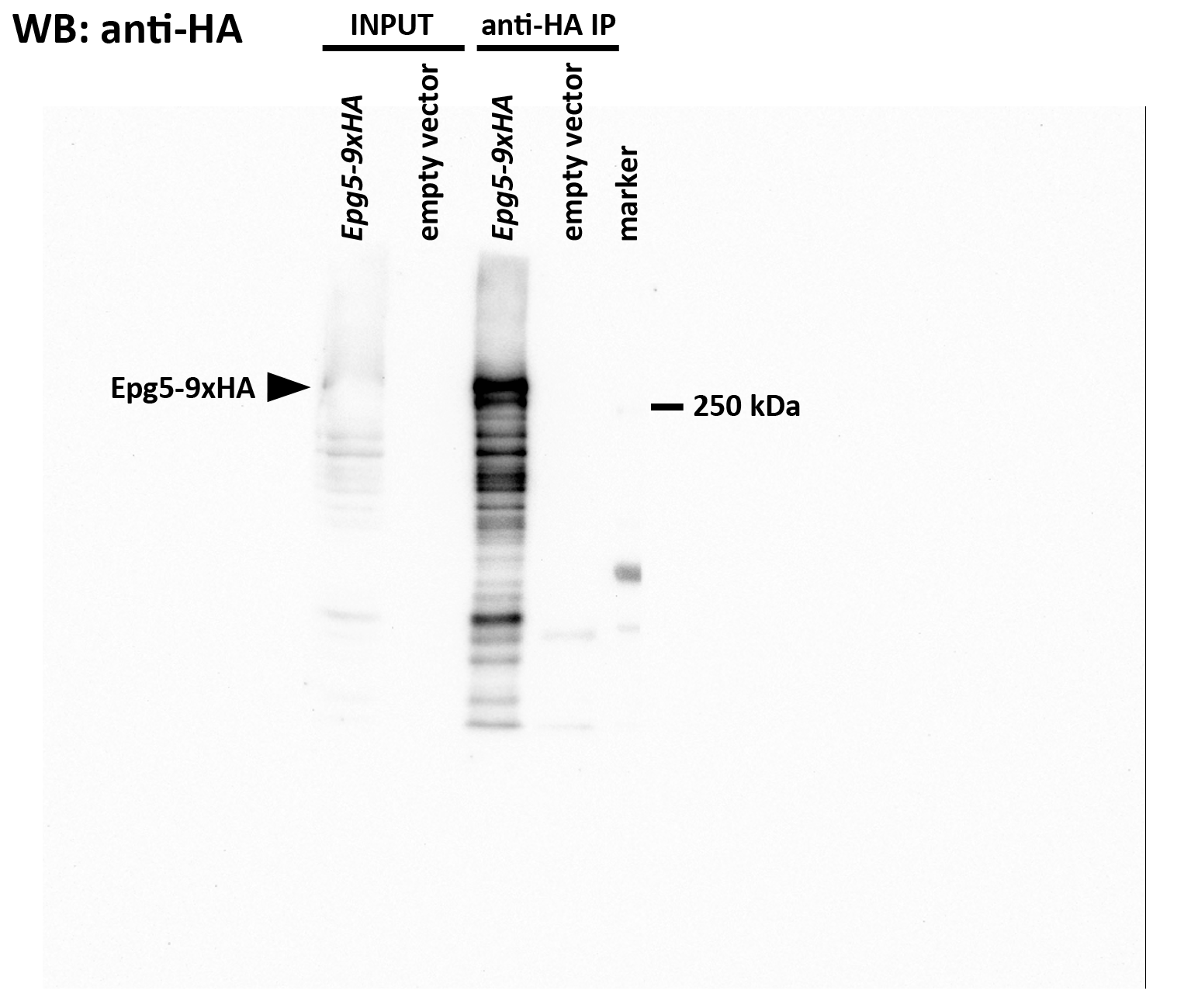

Supplement: Figure 5—source data 2. [file elife-102663-fig5-data2.zip › 5M_anti-HA_raw_labelled.tif]

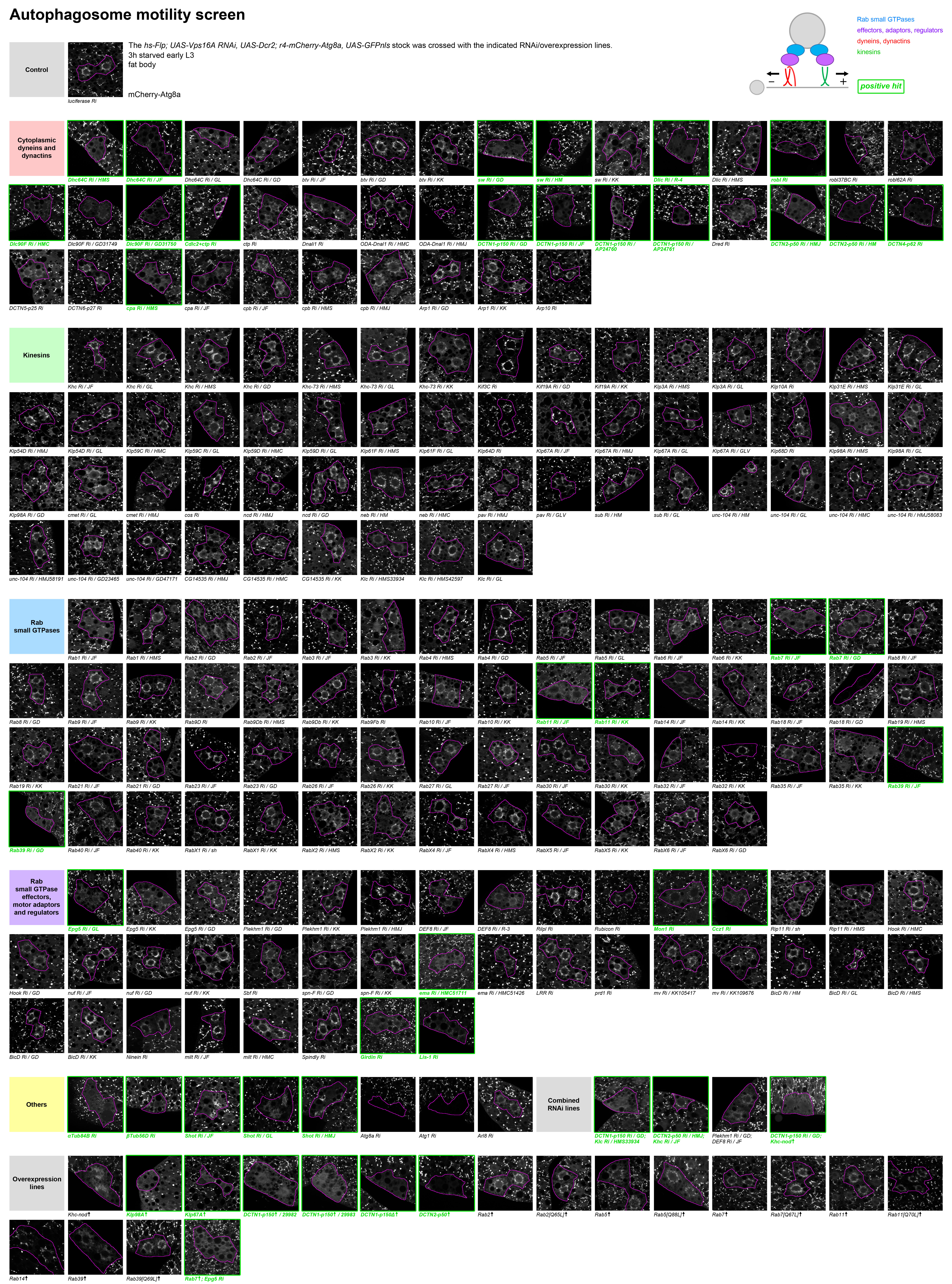

Supplement: Supplementary file 1. — The boundaries of silenced or overexpressing cells are highlighted in magenta, while positive hits are marked with green frames and captions. [file elife-102663-supp1.zip › Supplementary File 1.jpg]
